# The *Wolbachia* WO bacteriophage proteome in the *Aedes albopictus* C/*w*Str1 cell line: evidence for lytic activity?

**DOI:** 10.1007/s11626-015-9949-0

**Published:** 2015-10-01

**Authors:** Gerald D. Baldridge, Todd W. Markowski, Bruce A. Witthuhn, LeeAnn Higgins, Abigail S. Baldridge, Ann M. Fallon

**Affiliations:** Department of Entomology, University of Minnesota, 1980 Folwell Ave., St. Paul, MN 55108 USA; Department of Biochemistry, Molecular Biology and Biophysics, University of Minnesota, Minneapolis, MN 55455 USA; Feinberg School of Medicine, Northwestern University, Chicago, IL 60611 USA

**Keywords:** Mosquito cell line, Proteome, WO phage, Rickettsial plasmids

## Abstract

*Wolbachia pipientis* (*Rickettsiales*), an obligate intracellular alphaproteobacterium in insects, manipulates host reproduction to maximize invasion of uninfected insect populations. Modification of host population structure has potential applications for control of pest species, particularly if *Wolbachia* can be maintained, manipulated, and genetically engineered in vitro. Although *Wolbachia* maintains an obligate mutualism with genome stability in nematodes, arthropods can be co-infected with distinct *Wolbachia* strains, and horizontal gene transfer between strains is potentially mediated by WO phages encoded within *Wolbachia* genomes. Proteomic analysis of a robust, persistent infection of a mosquito cell line with *w*Str from the planthopper, *Laodelphax striatellus*, revealed expression of a full array of WO phage genes, as well as nine of ten non-phage genes that occur between two distinct clusters of WOMelB genes in the genome of *w*Mel, which infects *Drosophila melanogaster*. These non-phage genes encode potential host-adaptive proteins and are expressed in *w*Str at higher levels than phage structural proteins. A subset of seven of the non-phage genes is flanked by highly conserved non-coding sequences, including a putative promoter element, that are not present in a syntenically arranged array of homologs in plasmids from three tick-associated *Rickettsia* spp. These studies expand our understanding of *w*Str in a host cell line derived from the mosquito, *Aedes albopictus*, and provide a basis for investigating conditions that favor the lytic phase of the WO phage life cycle and recovery of infectious phage particles.

**Electronic supplementary material:**

The online version of this article (doi:10.1007/s11626-015-9949-0) contains supplementary material, which is available to authorized users.

## Introduction

*Wolbachia* (*Anaplasmataceae*; *Rickettsiales*) is an obligate intracellular alpha proteobacterium that engages in distinctive interactions with invertebrate hosts, depending on whether they are nematodes or arthropods. In nematodes, *Wolbachia* functions as a mutualist (Taylor *et al.*^2005^; Comandatore *et al.*^2013^) and is required for host survival. The genomes of nematode-associated *Wolbachia* supergroup C- and D-strains coevolve with their host genomes and lack mobile genetic elements that are abundant in strains from the arthropod-associated supergroups A and B, hereafter designated as WOL-A and WOL-B (Wu *et al.*^2004^; Cordaux *et al.*^2008^; Ishmael *et al.*^2009^; Newton and Bordenstein 2011). Arthropod-associated *Wolbachia* are typically vertically transmitted parasites that manipulate reproduction to invade uninfected populations of their hosts (Zug and Hammerstein 2014), but can also be transmitted horizontally to new host species by predators, parasites, and parasitoids (reviewed in Zug *et al.*^2012^). Arthropod hosts that are co-infected with multiple *Wolbachia* strains have provided an arena for genetic recombination, which is reflected in present-day strains by mosaic gene sequences, a lack of phylogenetic congruence between *Wolbachia* strains and arthropod hosts, and the presence of mobile genetic elements in *Wolbachia* genomes.

Arthropod-associated *Wolbachia* genomes typically contain one or more WO prophages consisting of a complete set of genes with a modular organization and encoding head, baseplate, tail, and all other proteins required for the lytic cycle and packaging of a potentially infectious phage (Kent *et al.*^2011^). WO prophages have been likened to lysogenic forms of bacteriophage lambda in *Escherichia coli* (Kent and Bordenstein 2010). Recombination or transposition near prophage termini is thought to be a major mechanism by which *Wolbachia* acquires DNA from other prokaryotic taxa (Ishmael *et al.*^2009^; Tanaka *et al.*^2009^; Kent *et al.*^2011^; Duplouy *et al.*^2013^). Foreign DNA associated with WO prophages encodes ankyrin repeat proteins, host cell adhesion and invasion factors, and type IV secretion system effectors, which have potential host-adaptive functions (Tanaka *et al.*^2009^; Kent *et al.*^2011^; Siozios *et al.*^2013^). In addition to the complete prophages typically present in WOL-A and WOL-B genomes, degenerate prophages are common and may occur in isolation as in ^A^*w*Sol (hereafter, supergroup designations are indicated by superscripts preceding the strain name) from the fig wasp, *Ceratosolen solmsi*, whose degenerate prophage has the highest known proportion (27.6%) of pseudogenes and lacks the tail module (Wang *et al.*^2013^). Genome reduction in *Wolbachia* may in fact target prophage sequences, as has been suggested for ^A^*w*Rec from *Drosophila recens* (Metcalf *et al.*^2014^).

Here, we define the prophage proteome from ^B^*w*Str, which maintains a robust infection in C/*w*Str1 mosquito cells. Proteomic analyses suggest that the ^B^*w*Str genome contains a prophage resembling WOMelB from the ^A^*w*Mel genome that infects *Drosophila melanogaster*. DNA sequence analyses verified detection of peptides corresponding to proteins encoded by a syntenic array of genes present in the ^B^*w*Str genome and in the WOMelB prophage as well as in plasmids from three *Rickettsia* spp. associated with ixodid ticks. Expression of proteins representing one or more complete prophages suggests that a lytic cycle occurs in C/*w*Str1 cells, which provide advantages of scale and ease of manipulation for future identification of conditions that favor isolation of infectious phage particles.

## Materials and Methods

### *Cell culture.*

Uninfected *Aedes albopictus* C7-10 and ^B^*w*Str-infected C/*w*Str1 cells were maintained in Eagle’s minimal medium supplemented with 5% fetal bovine serum at 28–30°C in a 5% CO_2_ atmosphere as described previously (Shih *et al.*^1998^; Fallon *et al.*^2013^).

### *Polymerase chain reaction, DNA cloning and sequencing, and sequence identity comparisons.*

The polymerase chain reaction (PCR) was used to amplify the ^B^*w*Str homologs of the ^A^*w*Mel loci WD0611–WD620 from template DNA prepared from *Wolbachia* enriched by fractionation of C/*w*Str1 cells by density gradient centrifugation (GF-50/60) as detailed elsewhere (Baldridge *et al.*^2014^). We obtained 21 PCR products using a panel of 69 primers (Table S1), GoTaq DNA polymerase (Promega, Madison, WI), and a Techne TC-312 cycler (Staffordshire, UK). Cycle parameters were 1 cycle at 94°C for 2 min, 35 cycles at 94°C for 35 s, 53°C for 35 s, 72°C for 1 min, followed by 1 cycle at 72°C for 5 min. Extension time was increased to 2 min for products ≥1000 bp. PCR products were cloned in the pCR4-TOPO vector with the TOPO-TA Cloning Kit for Sequencing (Life Technologies, Grand Island, NY), and two or more clones each were sequenced at the University of Minnesota BioMedical Genomics Center. DNA and protein alignments were executed with the Clustal Omega program (Sievers *et al.*^2011^). Alignments were edited and modified using Microsoft Word. All nucleotide and protein sequence identity comparisons were executed with the BLASTn and BLASTp algorithms available at http://blast.ncbi.nlm.nih.gov.

### *Mass spectrometry, peptide detection, and protein identification.*

LC–MS/MS on LTQ and Orbitrap Velos mass spectrometers was executed as described previously (Baldridge *et al.*^2014^). Tandem mass spectra were extracted by Sequest (Thermo Fisher Scientific, San Jose, CA; version SRF v.3 or version 27, rev. 12); Sequest parameters and protein sequence database information have been reported previously (see Table S4 in Baldridge *et al.*^2014^). Data were searched against an rs_wolbachia_aedes_v200808_cRAP_flavivirusREV database that contained 74,570 protein entries from sequenced *Wolbachia* genomes, the *Aedes aegypti* genome, and flavivirus genomes available as of July 2011. Assembled *Wolbachia* genomes included those of the ^B^*w*Pip WOL-B strain associated with *Culex pipiens quinquefasciatus* Pel mosquitoes from Sri Lanka (Klasson *et al.*^2008^), the *Drosophila*-associated WOL-A strains, ^A^*w*Mel (Wu *et al.*^2004^) and ^A^*w*Ri (Klasson *et al.*^2009^), and the nematode-associated WOL-D strain, ^D^*w*Bm (Foster *et al.*^2005^). Incomplete genomes included the *Drosophila*-associated ^A^*w*Ana and ^A^*w*Will WOL-A strains. Scaffold (version 4.2.1, Proteome Software Inc., Portland, OR) was used to validate MS/MS-based peptide and protein identifications. As detailed in the “Results,” the original MS search database was modified to support a refined search by inclusion of proteins deduced from sequenced ^B^*w*Str genes.

### *Relative abundance estimation and statistical analysis.*

RAL, or relative abundance level, is based on counts of unique peptides in four MS data sets, as shown for data sets MS-D, MS-E, MS-F, and MS-G in Table S2. In each column, values indicate number of peptides\% protein coverage (the percentage of amino acids in the full-length protein represented by MS peptides). As detailed in Baldridge *et al.* (2014), RAL scores indicate the relative abundance of a particular protein relative to the total of 790 *Wolbachia* proteins detected by mass spectrometry in C/*w*Str1 cells. For proteins grouped according to functional class, RAL scores ranged from a maximum of 7.7 to a minimum of 1.0 (see Table 4 in Baldridge *et al.*^2014^). SR, or studentized residuals, are derived from a statistical analysis of RAL scores normalized to protein mass and indicate deviance from expected values adjusted for estimated standard deviation from the mean. A protein of average abundance relative to all other *Wolbachia* proteins identified in C/*w*Str1 cells has an SR of 0, while above-average abundance is associated with a positive SR value and below-average abundance is associated with a negative value, with overall scores ranging from −2.36 to +3.69. The details of the statistical analysis are given in Baldridge *et al.* (2014). All tests of association were performed with SAS v9.3 (Cary, NC; http://www.sas.com/en_us/home.html/).

## Results

### *The WO phage proteome from the C/wStr1 cell line.*

We have previously shown that exponentially growing *A. albopictus* C/*w*Str1 cells express a ^B^*w*Str proteome of nearly 800 proteins, using a stringent threshold requiring detection of multiple peptides from the same protein within at least one of four MS data sets (Baldridge *et al.*^2014^). Based on those criteria, we identified 39 WO phage proteins, some of which were mosaic in the sense that they were represented by peptides corresponding to homologs from distinct *Wolbachia* genomes. As a group, these 39 phage/virus related proteins were expressed at relatively low abundance, with a relative abundance level (RAL) score of 1.5, compared to 7.7 for the most highly abundant functional group with protein modification/chaperone activities, and the lowest RAL of 1.0 for proteins of unknown function (Baldridge *et al.*^2014^). We attribute the aggregate low RAL score for phage-related proteins to our efforts to harvest ^B^*w*Str from cells in exponential growth phase. To a first approximation, these observations indicate that under optimal conditions for host cells, WO phage genes are expressed at detectable levels and possibly contribute to variation in *Wolbachia* levels among individual cells within a population.

Here, we examined WO phage expression more closely to determine whether the data supported expression of packaged phage. WO phage genes evolve rapidly (Kent *et al.*^2011^), and our stringent criteria for inclusion in the original ^B^*w*Str proteome therefore underestimated proteins encoded by prophage genomes. Re-examination of the original data including proteins represented by multiple homologs uncovered a final ^B^*w*Str prophage proteome of 119 proteins that included previously unreported proteins represented by single peptides (Tables 1 and S2). In aggregate, head (20), baseplate (9), and tail (10) proteins necessary for formation of viral particles accounted for 33% of the phage proteome, while 19 proteins (16%) have functions in DNA recombination (7 proteins), replication (5 proteins), or modification (7 proteins). An additional 19 proteins (16%) have known or likely roles in phage or *Wolbachia* interactions with host cells and virulence, while the remaining 41 proteins (34%) are homologs of WO phage proteins whose functions remain unknown. The prophage proteome included 16 proteins encoded by orphan genes in sequenced *Wolbachia* genomes and nine proteins encoded by foreign genes believed to have been acquired by a WO-B phage (Ishmael *et al.*^2009^; Kent *et al.*^2011^), seven of which occur as syntenic arrays on rickettsial plasmids. Thus C/*w*Str1 cells express proteins representing all modules and functional classes encoded by WO prophages in annotated genomes of representative *Wolbachia* strains, most of which correspond to intact prophages with potential lytic activity. For example, of 61 proteins that matched WO prophage genes in ^B^*w*Pip (Table S2), only four correspond to orphan genes.

**Table 1.** Functional classes of WO prophage proteins detected by LC–MS/MS in extracts of C/*w*Str1 cells

WO phage^a^	Locus range	Head	BP	Tail	REC	REP	MOD	VIR	UK	Total
MelA	0261–0288	2	2	–	1	–	1	1	3	10
MelB1	0565–0610	4	–	4	–	3	2	4	9	26
(Rick.)	0611–0620	–	–	–	–	–	–	5	4	9
MelB2	0633–0644	–	2	–	1	–	–	1	–	4
Pip1	0243–0272	–	–	–	1	1	–	1	5	8
Pip2	0297–0322	2	1	–	–	–	1	–	–	4
Pip3	0323–0342	3	1	–	–	–	–	2	1	7
Pip4	0411–0455	3	2	2	1	1	2	1	5	17
Pip5	1295–1340	3	1	3	–	–	1	2	7	17
Pip	Orphans	1	–	–	2	–	–	–	1	4
Mel	Orphans	–	–	–	–	–	–	1	4	5
Ana/Sim	Orphans	2	–	1	1	–	–	1	2	7
Total		20	9	10	7	5	7	19	41	119

^a^WO prophage sequences from WOMelA, WOMelB1, B2, and nine non-phage proteins with homologs on rickettsial plasmids (Rick), five WO phages in ^B^ *w*Pip, and various orphan phage genes. Structural proteins are defined as phage head, baseplate (BP), and tail modules. See Table S2 for complete list of proteins

*REC* proteins with recombinase/resolvase/integrase, and transposase activities, *REP* replication functions, *MOD* DNA methylase, endonuclease and SNF2 helicase functions, *VIR* ankyrin repeat, patatin family, gp15, and hypothetical proteins, *UK* unknown functions

### *Similarities to WOMelB and rickettsial plasmids.*

**Figure 1.**
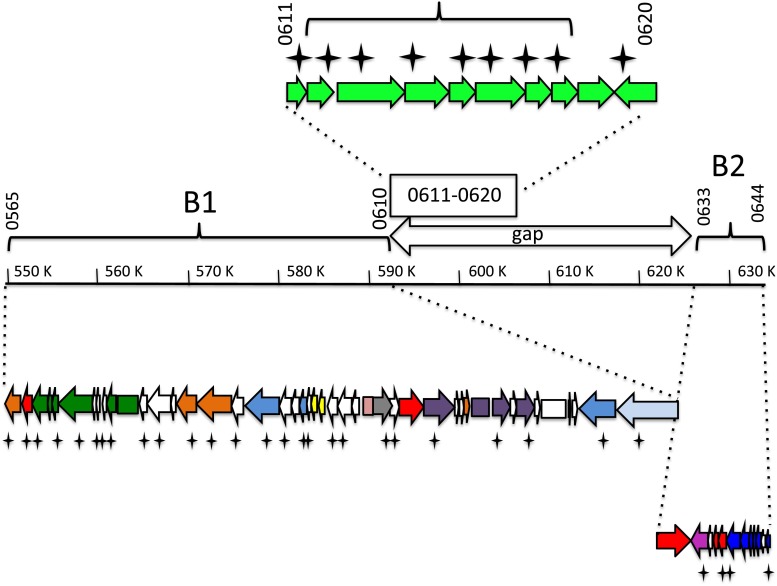
Schematic depiction of proteins in the ^B^wStr WO phage proteome matched to peptides from homologs encoded by the ^A^ *w*Mel WO-B prophage. As detailed by Kent *et al.* (2011), ^A^ *w*Mel WO-B occurs as two blocks of phage genes (B1 and B2) separated by ∼35 kb of non-phage DNA. The central schematic represents the complete WO-B phage extending from WD0565 to WD0644. The region labeled “gap” corresponds to 35 kb separating B1 and B2 that contains foreign genes (WD0611 to WD0620) shown at top as *light green arrows* representing open reading frames. The *bracket* indicates genes that occur as syntenic *arrays* of homologs on three rickettsial plasmids. At the *bottom*, open reading frames of genes in the B1 and B2 blocks are indicated by *color-coded arrows* oriented in the direction of transcription (phage head, *purple*; baseplate, *dark blue*; tail, *green*; recombinase, *pink*; replication, *light blue*; transposase, *yellow*; virulence factors, *orange* as depicted in Fig. 1 in Kent *et al.* 2011). *Stars* designate proteins matched to ^B^ *w*Str peptides.

The most abundantly represented ^B^*w*Str WO phage proteins corresponded to homologs encoded by WOMelB (Fig. 1), which are depicted schematically by crosses below arrows representing open reading frames. WOMelB is organized as two blocks of genes: B1: WD0565 to WD0610 and B2: WD0633 to WD0644, relative to a single block of genes in the degenerate WOMelA (WD0261 to WD0288; see Fig. 1 in Kent *et al.*^2011^). The 35-kb gap separating the B1 and B2 regions includes ten non-phage genes (WD0611–WD0620), of which nine were represented by ^B^*w*Str peptides. WD0612–WD0618 also occur in syntenic arrangement on rickettsial plasmids (see below). In addition, we recovered ^B^*w*Str peptides corresponding to ten proteins encoded by WOMelA and 12 proteins encoded by phage orphan genes in the ^A^*w*Mel, ^A^*w*Ana, and ^A^*w*Sim genomes (Tables 1 and S2). Overall, the WO phage peptides recovered in this analysis suggest that intact prophages similar to WOMelB (Fig. 1) and WOPip4/Pip5 (Tables 1 and S2) occur in the ^B^*w*Str genome. We note that the mean relative abundance levels (RAL; for details see Baldridge *et al.*^2014^) of proteins from potentially intact prophages range from 0.97 to 1.26 (Table S2), and are two to fourfold higher than those of degenerate prophage and orphan proteins (range 0.30–0.62). These expression levels, combined with detection of critical proteins such as the phage recombinase/integrase, RepA, and others involved in DNA replication, modification, and packaging, suggest that ^B^*w*Str encodes at least one prophage capable of excision and completion of the lytic life cycle.

### *Homologs of rickettsial plasmid genes.*

**Figure 2.**
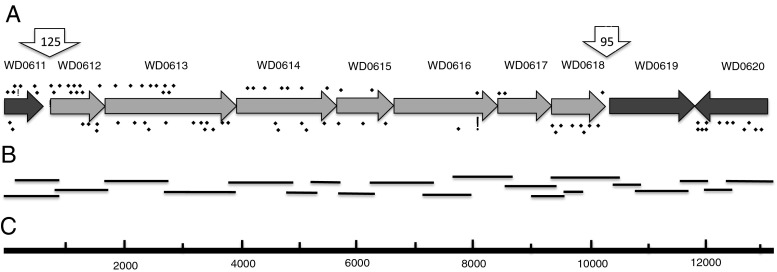
Schematic map of PCR-amplified ^B^ *w*StrB homologs of prophage-associated genes and genes on rickettsial plasmids. (*A*) The ^B^ *w*Str genes are depicted as *arrowheads* oriented in the direction of transcription. Genes are identified by WOMelB locus tags (Genbank Acc. # NC_002978.6). *Light shading* indicates genes present in both WO prophages and in plasmids from *Rickettsia* spp. *Downward arrows* indicate sites of conserved non-coding regions (see Fig. 3). The *diamond symbols* below and above the *arrows* indicate unique peptides identified in the original and refined searches of the MS data sets, respectively. See Table 3 for protein identities. (*B*) *Horizontal lines* represent cloned PCR amplification products. See Table S1 for primers. (*C*) Scale marker in 1-kb increments.

The phage-associated WD0611–WD0620 gene array in WOMelB (Fig. 1) is of special interest because it encodes proteins that may mediate host–microbe interactions and is conserved in other WO phages, such as ^A^wRi and ^A^wVitA2 (Kent *et al.*^2011^), and because homologs of WD0612–WD0618 occur as syntenic arrays on plasmids from *Rickettsia* spp. associated with ixodid ticks (see Table S3 for all accession numbers). We used PCR amplification and DNA sequencing to validate proteomic evidence for expression of WD0611–WD0620 homologs and to determine whether they occur as a block of contiguous genes in the ^B^*w*Str genome (Fig. 2 and Table S2, entries 51–59). In the ^B^*w*Str genome, a 13,127-bp sequence based on 21 overlapping PCR products (Fig. 2*B* and Table S3) begins near the 5′ end of the WD0611 homolog and ends 115 bp upstream of the WD0620 start codon (Fig. 2*A*). In addition to 53 unique peptides detected originally (Baldridge *et al.*^2014^) and represented by star symbols below the genes in Fig. 2*A*, the ^B^*w*Str sequence data resulted in detection of 42 new peptides (stars above genes) including 35 from WD0611–WD0616, three from the highly conserved WD0617 and WD0618, and four from the slightly less conserved WD0620. BLASTn comparisons (Table 2) confirmed that the WD0611–WD0620 genes from ^B^*w*Str have homologs in WOL-A and WOL-B genomes and that the WD0612–WD0618 genes have homologs on three rickettsial plasmids. These genes do not occur in nematode-associated WOL-C/D genomes, which lack prophages.

**Table 2.** Sequence identities of ^B^ *w*Str WD0611–620 homologs in *Wolbachia* strains and rickettsial plasmids

Gene^a^	^B^ *w*Bol1b		^B^ *w*VitB		^B^ *w*AlbB		^B^ *w*No		^A^ *w*Ha		^A^wAu		^A^ *w*Ri		^A^ *w*Mel		^A^ *w*Sol		^R^pReis2		^R^pRhe		^R^pREIP	
	Nt	AA	Nt	AA	Nt	AA	Nt	AA	Nt	AA	Nt	AA	Nt	AA	Nt	AA	Nt	AA	Nt	AA	Nt	AA	Nt	AA
WD0611^b^	*99*	*98*	*96*	*97*	*98*	*97*	*96*	*97*	85	79	85	80	85	79	85	80	85	79	–	–	–	–	–	–
WD0612	*99*	*97*	*98*	*97*	*98*	*98*	*98*	*97*	91	89	91	89	91	89	91	89	91	89	76	68	75	68	76	68
WD0613	*99*	*97*	*96*	*94*	*98*	*95*	*96*	*94*	90	91	90	90	90	90	90	90	90	90	78	76	78	75	78	76
WD0614	*98*	*97*	*98*	*97*	*97*	*97*	88	85	88	85	88	85	88	86	88	85	88	86	71	62	70	61	71	63
WD0615^b^	*98*	*97*	*99*	*99*	*98*	*97*	90	89	90	89	90	90	90	89	90	89	90	89	71	63	72	63	71	62
WD0616^c^	*98*	*99*	*99*	*99*	*97*	*97*	92	92	92	92	92	92	92	92	92	92	92	90	71	64	70	63	72	64
WD0617^c^	*98*	*99*	*99*	*99*	*97*	*99*	*99*	*99*	*99*	*99*	*99*	*99*	*99*	*99*	*99*	*99*	90	*94*	79	79	78	78	78	80
WD0618	*99*	*99*	*99*	*99*	*99*	*99*	*99*	*99*	*99*	*99*	*99*	*99*	*99*	*99*	*99*	*99*	90	90	75	73	75	72	75	73
WD0619	*96*	*96*	*97*	*97*	*97*	*97*	*98*	*96*	*98*	*98*	*97*	*97*	*98*	*98*	*98*	*98*	91	91	–	–	–	–	–	–
WD0620	*98*	*98*	*98*	*98*	*99*	*98*	*98*	*98*	*97*	*97*	*96*	*96*	*97*	*97*	*97*	*97*	91	88	–	–	–	–	–	–
125 NC^d^	*97*	nc	*98*	nc	*98*	nc	*97*	nc	83	nc	83	nc	83	nc	82	nc	83	nc	–	–	–	–	–	–
95 NC^d^	*100*	nc	*100*	nc	*99*	nc	*99*	nc	*100*	nc	*99*	nc	*100*	nc	*98*	nc	89	nc	–	–	–	–	–	–

Nucleotide (Nt) and amino acid (AA) identities (%) based on BLAST (NCBI) comparisons to ^B^ *w*Str homologs. Values in italic designate ≥94% identity. Host associations: ^B^ *w*VitB, wasp *Nasonia vitripennis*; ^B^ *w*AlbB, mosquito *Aedes albopictus*; ^B^ *w*Bol1b, butterfly *Hypolimnas bolina*; ^B^ *w*No, ^A^ *w*Ha, ^A^ *w*Ri, ^A^wAu, fly *Drosophila simulans*; ^A^ *w*Mel, fly *D. melanogaster*; ^A^ *w*Sol, fig wasp *Ceratosolen solmsi*; ^R^pReis2, rickettsial endosymbiont of *Ixodes scapularis* (Gillespie *et al.* 2012); ^R^pREIP, rickettsial endosymbiont of *Ixodes pacificus* (R. Felsheim, accession # KR611317); ^R^pRhe, *Rickettsia helvetica* from *I. ricinus* (Dong *et al.* 2012)

^a^Genes are identified by *w*Mel locus tags. See Table S3 for accession numbers. *Superscripts A*, *B*, and *R* indicate *Wolbachia* supergroups A and B and rickettsial plasmids, respectively. *Dashes* indicate genes that are absent in rickettsial plasmids; nc indicates non-coding sequences

^b^Partial sequences in ^B^ *w*VitB

^c^Premature stop codons in ^A^ *w*Sol, which contains a single degenerate prophage (Wang *et al.* 2013)

^d^Sequences that flank WD0612–0618 (see Fig. 2)

### *Sequence comparisons between Wolbachia strains.*

Nucleotide identities of the ^B^*w*Str WD0611–WD0616 homologs to WOL-B genes ranged from 96 to 99% (Table 2; italicized values), with the exception of WD0614–WD0616 in ^B^*w*No, which fell into the range (85–92%) of WOL-A strains. Pairwise comparisons between ^A^*w*Ha and ^B^*w*No homologs were 99% identical, suggesting that ^B^*w*No has acquired the WOL-A genes through genetic exchange during co-infection, which occurs in some populations of *Drosophila simulans* (James *et al.*^2002^). In contrast to the differential pattern of strain-related identities of WD0611–WD0616, WD0617–WD0620 were 96–99% identical among all strains with the exception of the degenerate prophage from ^A^*w*Sol, in which WD0616 and 0617 are pseudogenes. Finally, deduced amino acid identities were similar to nucleotide identities for all genes except WD0611, where values were 5–6% lower in WOL-A strain comparisons. We noted that WOL-A homologs were slightly longer and contained conserved amino acid substitutions relative to WOL-B homologs. These differences may reflect differential selection pressures on WD0611 versus other genes in WOL-A versus B strains.

### *Sequence comparisons between*^*B*^*wStr and rickettsial plasmids.*

^B^*w*Str WD0612–0618 nucleotide identities to homologs from three rickettsial endosymbionts of *Ixodes* ticks ranged from 70 to 80% (Table 2), with the internal genes WD0614, 0615, and 0616 having the lowest identities. Concatenated sequences of WD0612–0618 from the three plasmids shared 95–96% identity, and pairwise comparisons of individual pREIS2 genes to those from the representative WOL-A (^A^*w*Ha) and WOL-B (^B^*w*Str) strains showed differences of ≤1% among the rickettsial plasmid genes. In comparisons to the ^B^*w*Str sequences, the rickettsial WD0612, 0614, 0615, and 0616 homologs had deduced amino acid identities that were 7–9% lower than nucleotide identities versus only 0–3% lower in WD0613, 0617, and 0618 comparisons.

### *Conserved sequences flanking the Wolbachia WD0612*–*0618 homologs contain potential transcriptional regulatory elements*.

**Figure 3.**
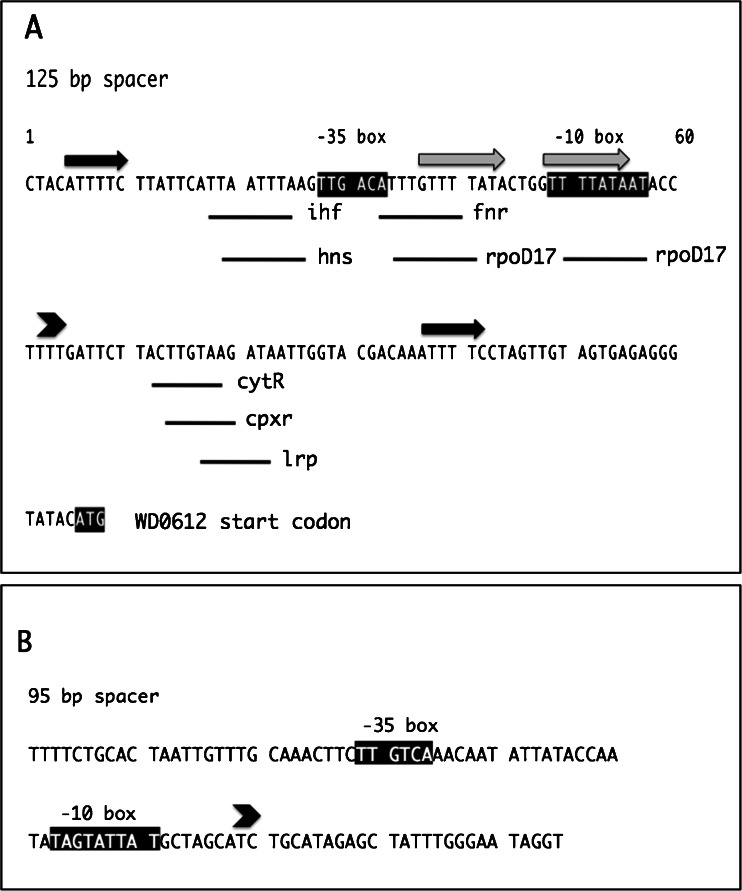
Non-coding sequences in the WD0611–WD0620 syntenic array. Sequences of the non-coding 125-bp region upstream of WD0612 (*A*) and the 95-bp region downstream of WD0618 (*B*) were annotated using Softberry BPROM prediction (http://linux1.softberry.com/berry.phtml?topic=bprom&group=programs&subgroup=gfindb; Solovyev and Salamov 2011). In *A* and *B*, the *–35* and *–10 boxes* associated with predicted promoters are indicated in *white font* with *black* shading, and an *arrowhead* above the sequence marks the promoter site. (*A*) *black* and *gray horizontal arrows* above the sequence indicate two sets of direct repeats; positions of DNA-binding protein and transcription factor interaction sites described in the “Results” are indicated by *bars* below the sequence. The ATG start site for WD0612 is indicated in *white font* with *black shading*. (*B*) No predicted transcription factor binding sites occur in the 95-bp sequence.

In ^B^*w*Str, the WD0612–WD0618 homologs were arranged as directly adjacent or overlapping ORFs, suggesting possible organization as an operon. At the 5′ end, WD0612 was separated from the upstream WD0611 by 125 bp, and at the 3′ end, WD0618 was separated from WD0619 by 95 bp (Fig. 2*A*). These flanking sequences had no significant BLAST similarities to one another and were not self-complementary. The 125-bp non-coding sequence (Fig. 3) is highly conserved among WOL-B strains (97–98% nucleotide identities) but less so (range 82–83%) with WOL-A strains (Table 2). The ^B^*w*Str 125-bp sequence contains two short direct repeats, a –35 box and –10 box in a potential prokaryotic promoter, and interaction sites for several *E. coli* DNA-binding proteins and transcription factors (Fig. 3*A*), including ihf, involved in bacteriophage lambda integration and recombination; hns, bacterial chromosome organization and global modulation of gene expression; fnr, an oxygen-responsive regulator; rpoD17, a “housekeeping” sigma factor that interacts with RNA polymerase; cytR, involved in nucleoside uptake and metabolism; cpxR, involved in expression of outer membrane porin proteins; and lrp, a metabolic regulator involved in amino acid metabolism. Although the presence of these potential transcriptional regulatory elements is intriguing, their functional context is unclear because the WD0612–WD0618 proteins are expressed at variable levels and represent a diverse array of potential functions. Moreover, the highly reduced genomes of *Wolbachia* strains encode a small suite of known transcription factors relative to *E. coli*, and their functions are largely unexplored. The ^B^*w*Str 95-bp sequence (Fig. 3*B*) following the 3′ end of WD0618 is also highly conserved (98–100% identity) among all ten *Wolbachia* strains except the degenerate ^A^*w*Sol (89%). Although the sequence has a potential promoter, it lacks transcription factor binding sites. Uniquely, the consecutive gene, WD0619, was not represented in the ^B^*w*Str proteome, while WD0620, encoded on the opposite strand, was represented by 12 peptides (Fig. 2*A*).

### *Relative abundance of WD0611*–*WD0620 homologs in C/wStr1 cells.*

With the exception of WD0619, for which no ^B^*w*Str peptides were recovered, protein coverage by MS/MS detected peptides from WD0611–WD0620 homologs ranged from 5 to 58% (Table 3). We estimated protein relative abundance levels (RAL) using studentized residuals (SR), a measure of deviance from expected values adjusted for estimated standard deviation from the mean as reported in Table S4, in which the WD0611–0620 homologs are in blue font. WD0612 and WD0613 were the most abundantly expressed, while WD0614, WD0615, and WD0616 were among the least abundant (Table 3).

**Table 3.** Relative abundance of WD0611–0620 protein homologs in ^B^ *w*Str

Locus^a^	kDa^b^	Pep.^c^	Cov.^d^	RAL	SR	Predicted protein
WD0611	28	8	36	4.0	0.20	UDP-*N*-acetylglucosamine pyrophosphorylase
WD0612	36	14	58	8.0	1.11	NAD-dependent epimerase/dehydratase
WD0613	90	22	31	10	0.49	Glycosyl transferase (group 1) moaA/nifB/pqqE family protein
WD0614	65	12	28	4.5	−0.47	Hypothetical with *O*-methyltransferase
WD0615	37	5	22	3.2	−0.37	Hypothetical with phytanoyl-CoA dioxygenase domain
WD0616	67	2	5	0.5	−2.68	ABC transporter, permease/ATP-binding protein
WD0617	39	12	42	3.8	−0.27	l-*allo*-Threonine aldolase
WD0618	39	9	34	3.5	−0.02	l-*allo*-Threonine aldolase
WD0619	44	0	0	0	–	GlpT/PgpT/UhpT transporter family protein
WD0620	48	12	33	3.5	−0.02	UDP-glucose 6-dehydrogenase

For details, see Baldridge *et al.* (2014)

*RAL* relative abundance level based on counts of unique peptides; *SR* studentized residuals derived from a statistical analysis of parameters that contribute to RAL scores

^aA^ *w*Mel locus tag

^b^Protein mass in kilodaltons

^c^Number of unique 95% confidence peptides detected by LC–MS/MS

^d^Percent coverage of amino acid sequence

WD0611 encodes a UDP-*N*-acetylglucosamine pyrophosphorylase, with slightly above-average abundance (RAL = 4.0; SR = 0.20). The enzyme contains N- and C-terminal domains with uridyl- and acetyltransferase activities and catalyzes the final steps in synthesis of UDP-*N*-acetylglucosamine, which is an essential precursor in lipopolysaccharide metabolic pathways. Although *Wolbachia* does not synthesize a cell wall, it expresses all proteins necessary for lipid II synthesis, which is required for cell division (Vollmer *et al.*^2013^). WD0612 encodes an abundant NAD-dependent epimerase/dehydratase (RAL = 8.0; SR = 1.11) that is a member of the WcaG group of proteins (COG0451) involved in lipopolysaccharide biosynthesis, bacterial cell envelope biogenesis, and modification of surface carbohydrates. WD0612 from ^B^*w*Str has 58% BLASTp identity to UDP-glucose 4-epimerase from *Chondromyces apiculatus* (EYF00501.1; Table 4), and its probable role in cell surface polysaccharide modification may intersect with that of the slightly below-average abundance UDP-glucose 6-dehydrogenase (WD0620; RAL = 3.5; SR = −0.02).

**Table 4:** High-scoring BLASTp hits to WD0611–0620 homologs from *Wolbachia* strain ^B^ *w*Str

Locus^a^	BLASTp hit description	Organism	Cover.^b^	Eval.^c^	Ident.^d^	Accession
WD0611	UDP-*N*-acetylglucosamine pyrophosphorylase	***Wolbachia strain wMel***	85%	4e−126	80%	AAS14312.1 GI:42410202
	Glucosamine-1-phosphate *N*-acetyltransferase	*Anaplasma marginale*	86%	2e−31	44%	WP_037330867.1 GI:739470965
	*N*-Acetylglucosamine uridyl/acetyltransferase	*Roseivivax halodurans*	83%	9e−27	35%	WP_037262448.1 GI:739401896
	*N*-Acetylglucosamine uridyl/acetyltransferase	*Halocynthiibacter sp. PAMC 20958*	82%	1e−26	37%	WP_039000580.1 GI:742871522
WD0612	NAD-dependent epimerase/dehydratase	***Wolbachia strain wMel***	100%	0	89%	AAS14313.1 GI:42410203
	Hypothetical protein, partial	*Burkholderia andropogonis*	89%	4e−79	45%	WP_036011746.1 GI:738052857
	NAD-dependent dehydratase	*Chondromyces apiculatus*	91%	4e−75	42%	WP_044251156.1 GI:763394096
	NAD-dependent epimerase/dehydratase	*Desulfovibrio hydrothermalis*	91%	3e−65	39%	YP_007324352.1 GI:436839974
	NAD-dependent epimerase/dehydratase	*Roseobacter sp. SK209*–*2*–*6*	89%	3e−65	41%	EBA16113.1 GI:126719404
WD0613	Glycosyl transferase (group 1) moaA/nifB/pqqE	***Wolbachia strain wMel***	100%	0	90%	AAS14314.1 GI:42410204
	Glycosyl transferase family 1	*Haliangium ochraceum*	99%	0	51%	WP_012826022.1 GI:502588332
	Glycosyl transferase group 1	*Desulfovibrio hydrothermalis*	99%	0	50%	WP_015334857.1 GI:505147755
	Hypothetical protein	*Capnocytophaga granulosa*	99%	0	47%	WP_016419668.1 GI:512455643
	Glycosyl transferase family 1	*Stigmatella aurantiaca*	51%	1e−137	50%	WP_002615805.1 GI:488691655
	Glycosyl transferase family 1	*Saprospira grandis*	55%	1e−133	49%	WP_015691633.1 GI:505729658
WD0614	Hypothetical protein	***Wolbachia strain wMel***	99%	0	85%	AAS14315.1 GI:42410205
	Methyltransferase	*Thiomonas sp. FB-Cd*	94%	2e−81	32%	WP_031406898.1 GI:670458099
	*O*-Methyltransferase family 2	*Desulfovibrio hydrothermalis*	96%	1e−79	33%	WP_015334858.1 GI:505147756
	*O*-Methyltransferase	*Saprospira grandis*	41%	8e−58	45%	WP_015691628.1 GI:505729653
	Hypothetical protein	*Capnocytophaga granulosa*	41%	9e−56	45%	WP_016419671.1 GI:512455646
WD0615	Hypothetical protein	***Wolbachia strain wMel***	100%	0	89%	AAS14316.1 GI:42410206
	Biosynthesis mitomycin/polyketide fumonisin	*Candidatus Regiella insecticola*	96%	2e−59	36%	WP_006705824.1 GI:493756998
	Phytanoyl-CoA dioxygenase	*Haliangium ochraceum*	65%	53–14	28%	YP_003265415.1 GI:262194206
	Phytanoyl-CoA dioxygenase	*Leisingera aquimarina*	67%	1e−09	28%	WP_027257567.1 GI:653005398
	Phytanoyl-CoA dioxygenase	*Rhodobacterales bacterium Y4I*	66%	1e−08	27%	WP_008553226.1 GI:495828647
WD0616	ABC transporter, permease/ATP-binding	***Wolbachia strain wMel***	100%	0	92%	AAS14317.1 GI:42410207
	ABC transporter permease	*Orientia tsutsugamushi*	98%	0	50%	WP_012461184.1 GI:501437567
	ABC transporter permease	*Cand. Paracaedibacter symbiosus*	98%	0	47%	WP_032112167.1 GI:692231795
	ABC transporter permease	*Cand. Paracaedibacter acanthamoebae*	97%	3e−169	45%	WP_038463806.1 GI:740678517
	ABC transporter permease	*Francisella sp. W12*–*1067*	97%	3e−142	43%	WP_035721422.1 GI:737752813
	Hypothetical protein	*Cand. Amoebophilus asiaticus*	97%	2e−137	41%	WP_012473081.1 GI:501449632
WD0617	l-*allo*-Threonine aldolase	***Wolbachia strain wMel***	100%	0	99%	AAS14318.1 GI:42410208
	Threonine aldolase	*Bacillus thuringiensis*	98%	4e−108	48%	WP_042332508.1 GI:754976739
	Threonine aldolase	*Anoxybacillus flavithermus*	98%	4e−104	47%	WP_032099946.1 GI:692183676
	Threonine aldolase	*Bacillus subtilis*	97%	2e−103	46%	KFF55387.1 GI:671703452
	Threonine aldolase	*Geobacillus kaustophilus*	98%	3e−103	48%	WP_011232238.1 GI:499551455
WD0618	l-*allo*-Threonine aldolase	***Wolbachia strain wMel***	98%	0	99%	AAS14319.1 GI:42410209
	Low-specificity l-threonine aldolase	*Halanaerobium saccharolyticum*	98%	3e−97	45%	WP_005489111.1 GI:491631573
	threonine aldolase	*Cand. Regiella insecticola*	96%	3e−96	48%	EGY27736.1 GI:347602761
	Threonine aldolase	*Nitrosococcus halophilus*	98%	9e−96	44%	WP_013034649.1 GI:502799673
	Threonine aldolase	*Halanaerobium hydrogeniformans*	100%	4e−92	44%	WP_013406697.1 GI:503172036
WD0619	GlpT/PgpT/UhpT transporter family protein	***Wolbachia strain wMel***	100%	0	97%	AAS14320.1 GI:42410210
	Hypothetical protein	*Cand. Paracaedibacter acanthamoebae*	96%	5e−41	30%	WP_038467018.1 GI:740681729
	sn-Glycerol-3-phosphate transporter	*Bacillus cereus*	91%	1e−33	28%	WP_016716465.1 GI:515087114
	sn-Glycerol-3-phosphate transporter	*Vibrio tasmaniensis*	94%	2e−33	26%	WP_029189257.1 GI:656242627
	MFS transporter	*Vibrio cyclitrophicus*	94%	3e−33	26%	WP_029189257.1 GI:656242627
WD0620	UDP-glucose 6-dehydrogenase	***Wolbachia strain wMel***	100%	0	97%	AAS14321.1 GI:42410211
	UDP-glucose 6-dehydrogenase	*Clostridium josui*	97%	2e−129	44%	WP_034847661.1 GI:736847311
	UDP-glucose 6-dehydrogenase	*Rhizobium tropici*	97%	2e−127	44%	WP_015340869.1 GI:505153767
	UDP-glucose 6-dehydrogenase	*Microvirga lupini (Rhizobiales)*	96%	3e−126	44%	WP_036370866.1 GI:738419381
	UDP-glucose 6-dehydrogenase	*Bradyrhizobium diazoefficiens USDA 110*	97%	1e−125	44%	NP_774769.1 GI:27383240

High-scoring BLASTp hits other than those in the genera *Wolbachia* and *Rickettsia* of the *Rickettsiaceae*

^aA^ *w*Mel locus tag

^b^Percent sequence coverage designates the proportion of the complete protein sequence recovered as individual peptides by mass spectrometry

^c^ *E* value

^d^Percent identity are based on BLASTp scores

WD0613 (RAL = 10; SR = 0.49) is particularly interesting because it contains a glycosyl transferase type 1 ExpE7-like domain (cd03823) in the N-terminal half of the protein. In *Sinorhizobium meliloti*, ExpE7 is involved in biosynthesis of galactoglucan exopolysaccharide II (Becker et al., 1997), which facilitates plant host cell adhesion and invasion, provides anti-oxidant defense, and may modulate plant defense responses during bacterial colonization (Lehman and Long 2013). The C-terminal half of WD0613 contains a superfamily domain (cd01335) typical of enzymes that use a molecule of *S*-adenosylmethionine in close proximity to 4Fe-4Fs iron–sulfur clusters to generate a deoxyadenosyl radical. A SPASM domain (pfam13186) located near the C-terminus provides an additional binding site for iron–sulfur clusters. These domains often occur in enzymes involved in biosynthesis of vitamins, coenzymes, and antibiotics or modification of other proteins. WD0613 is thus a potential multi-functional protein that may influence *Wolbachia* host interactions through modification of membrane proteins and surface interactions or supplementation of host cells with vitamins or coenzymes.

SR values indicate that WD0614 and 0615 have below-average abundances in C/*w*Str1 cells. WD0614 contains an unusual fusion of an N-terminal KWG *Leptospira* repeat domain (pfam14903) to an *O*-methyltransferase type 2 domain (Gillespie *et al.*^2012^). In many prokaryotes, methylation of DNA protects against degradation by restriction enzymes, which have not been identified in *Wolbachia*. WD0615 is a phytanoyl-CoA dioxygenase, which catalyzes conversion of alpha-ketoglutarate and phytanoyl-CoA to succinate and 2-hydroxyphytanoyl-CoA. In oceanic cyanobacteria, generation of succinate by a cyanophage-borne phytanoyl-CoA dioxygenase was suggested to play a role in energy generation under nutritional stress conditions (Sullivan *et al.*^2010^).

WD0616 (SR = −2.68), an ABC transporter permease/ATP binding protein with a possible role in multidrug efflux and/or iron–sulfur cluster transport, is one of the least abundant proteins in the ^B^*w*Str proteome. The WD0619 GlpT/PgpT/UhpT transporter with a predicted function in phosphoglycerate uptake, if expressed, was below the limit of detection by LC–MS/MS. We note that the majority of transporter proteins in the ^B^*w*Str proteome were of below-average abundance (SR < 0, Table S4, Baldridge *et al.*^2014^).

Two l-allo-threonine aldolases (WD0617 and 0618) with below-average abundances interconvert l-3-hydroxy-α-amino acids to glycine and an aldehyde in reactions similar to those catalyzed in ^B^*w*Str by an abundant serine hydroxymethyltransferase (Baldridge *et al.*^2014^). WD0617 and 0618 share only 31% amino acid identity and may be specialized for different and as yet unknown functions (Contestabile *et al.*^2001^; diSalvo *et al.*^2014^).

Although BLASTn comparisons indicated insufficient sequence identities to support broad phylogenetic analyses of WD0611–WD0620 from ^B^*w*Str, BLASTp comparisons suggest that homologs may be present in distantly related bacteria (Table 4). To evaluate these relationships, ^A^*w*Mel values are in bold as representative *Wolbachia* reference values. Species in which two or more syntenically arranged homologs may occur are underlined. These include marine sulfate-reducing *Desulfovibrio hydrothermalis* (WD0612–0614), for which BLASTp comparisons indicated sequence coverages ≥91% with identities that ranged from 33 to 50%. Similarly, genomes from the chemoautotroph *Haliangium ochraceum*, *Capnocytophaga granulosa*, and *Saprospira grandis* encode potential syntenically arranged homologs of WD0613, WD0614, and/or WD0615. Among all of the proteins, sequence coverages, *E* values ≤1e−125, and percent identities ≥44% indicated that WD0613 followed by WD0616 and WD0620 were most similar to proteins from other taxa.

## Discussion

In the absence of a ^B^*w*Str genome, the proteomics approach described here provides strong evidence for expression of WO phage genes in C/*w*Str cells harboring relatively high levels of *Wolbachia* (Baldridge *et al.*^2014^). Because *Wolbachia* abundance is inversely correlated with increasing WO phage densities in the wasp *Nasonia vitripennis* (Bordenstein *et al.*^2006^; Bordenstein and Bordenstein 2011), it will be of interest to learn whether phage activity can be induced to higher levels in C/*w*Str1 cells. Detection of peptides corresponding to the phage-related WD0611–WD0620 homologs with potential host-adaptive functions (Kent *et al.*^2011^) further supports the presence of one or more active WO phages in ^B^*w*Str. Sequenced *Wolbachia* genomes indicate that phage-associated host-adaptive functions potentially involve proteins with ankyrin repeat domains (Table S2, entries 15, 23, 65, 72, 93, 94, 123, 144) that may mediate protein–protein interactions (Pan *et al.*^2008^; Siozios *et al.*^2013^). Other proteins with likely roles in phage infectivity or host interactions include patatin-like phospholipases (Table S2, entries 22 and 114) known to be involved in rickettsial infection of host cells (Rahman *et al.*^2013^), as well as homologs of the VrlC virulence-related protein from the sheep pathogen, *Dichelobacter nodosus* (entries 32, 33 and 128), that were first identified in WOCauB2/B3 (Tanaka *et al.*^2009^). Finally, we note that a single unique peptide matched a “phage host specificity protein” (Table S2, entry 152) that contains domains found in a small phage-like particle containing DNA that is released from *Rhodobacter capsulatus* cells (Leung *et al.*^2010^).

Although annotation of sequenced *Wolbachia* genomes provides firm evidence for potentially active WO phages, physical detection of viral particles associated with *Wolbachia* has been achieved in only a few instances. Transmission electron micrographs of ovaries from *Culex pipiens* mosquitoes provided the first evidence for association of *Wolbachia* with particles resembling bacteriophages (Wright *et al.*^1978^). More than 20 yr later, Masui *et al.* (2000) characterized phage-related sequences in a DNA library from ^B^*w*Tai, which infects the cricket *Teleogryllus taiwanemma*. Hybridization of a probe from *orf*7, which has homology to a lambda minor capsid protein, to DNA from each of eight *Wolbachia* genomes, suggested that WO phages are widespread. RT-PCR experiments further showed that *orf*7 was expressed, and phylogenetic comparisons of *orf*7 sequences indicated that WO phages have mosaic structures suggestive of horizontal transmission within a common pool of double-stranded DNA phages (Masui *et al.*^2000^). Gavotte *et al.* (2004) extended these observations, using *orf*7 to identify WO phages in additional *Wolbachia* strains, and in some cases observed phage particles captured on 0.22-μm filters. Completion of the ^A^*w*Mel genome, and annotation of WOMelA and WOMelB, provided the basis for characterization of prophage sequences in a wide range of *Wolbachia* genomes (reviewed in Metcalf and Bordenstein 2012).

The WO phage is believed to be similar to the *E. coli* bacteriophage lambda (Tanaka *et al.*^2009^; Kent and Bordenstein 2010), and Bordenstein and Bordenstein (2011) have shown that in the wasp, *N. vitripennis*, temperature influences the balance between lysogenic/lytic development of the WO phage, *Wolbachia* densities, and cytoplasmic incompatibility. It will be of interest to investigate whether heat or cold treatments that increase phage abundance in *N. vitripennis* can be adapted to C/*w*Str1 cells to optimize recovery of WO phage particles, which may be facilitated by advantages of scale provided by cell culture. We are encouraged by the successful isolation of ^B^*w*CauB from gram quantities of host insects, without enrichment of infected tissues by dissection (Fujii *et al.*^2004^; Tanaka *et al.*^2009^). Successful isolation of physical particles will allow direct sequencing of ^B^*w*Str WO phage. Finally, we note that efforts to transform *Wolbachia* with transposon and plasmid-based technologies developed for related *Rickettsiales* genera (Felsheim *et al.*^2006^, ^2010^; Baldridge *et al.*^2007^; Burkhardt *et al.*^2011^) have been unsuccessful, further underscoring the need to investigate phage-mediated genetic exchange as a means to facilitate genetic manipulation of *Wolbachia* (Metcalf and Bordenstein 2012).

## Electronic supplementary material

Supplemental Table S1 Polymerase chain reaction (PCR) primers and amplification products from ^B^ *w*Str homologs of WD0611–WD0620. Products are depicted schematically in Fig. 2b. (DOCX 116 kb)

Supplemental Table S2 LC-MS/MS detection of 95% confidence unique peptides matched to proteins encoded by WO prophage and prophage-related genes. Columns A and B show WO prophage-encoded proteins and locus tags (as identified by Kent *et al.* 2011) as well as total numbers and proportions of encoded proteins matched to unique peptides in MS/MS data sets D, E, F and G in corresponding columns. Total number of matched peptides and percent protein sequence coverage are reported in columns D to G; e.g., 2/5 indicates 2 peptides constituting 5% protein sequence coverage. Column H reports the mean number of detected peptides, a rough estimate of the protein’s relative abundance level (RAL), which can be normalized to expected values for all proteins of that same mass (see SR values in Tables S3 and S4) as described by Baldridge *et al.* 2014. (XLSX 30 kb)

Supplemental Table S3 Genbank accession numbers for PCR-amplified and cloned *w*Str genes and homologs from other *Wolbachia* strains. (XLS 271 kb)

Supplemental Table S4 Results of Univariable and Multivariable Analyses after log transformation of the outcome, Peptide Count, and predictor, Molecular Weight. This table reports results for the original (Baldridge *et al.* 2014), and refined searches of the LC-MS/MS data sets D, E, F and G. See tabs at bottom: Sheet 1 reports mean SR values for all proteins in the original and refined searches in columns M and R. Runs 1, 2, 3 and 4 correspond to MS data sets D, E, F and G, respectively; Univariable Model and Multivariable Model (adjusted for functional class and MS Dataset) for results of tests of association. Proteins encoded by cloned genes from ^B^ *w*Str reported in this manuscript (WD0611 – WD0620 homologs) are in blue font and the ♦ symbols below and above the corresponding arrowheads in Fig. 2 indicate unique peptides identified in the original and refined searches of the MS data sets, respectively. Proteins encoded by cloned genes from ^B^ *w*Str reported in a previous refined search (Baldridge et al., submitted *Archs Microbiol*) are highlighted in yellow. (XLSX 24 kb)
