# Supplementary material for: The Wolbachia WO bacteriophage proteome in the Aedes albopictus C/wStr1 cell line: evidence for lytic activity?
Source: In Vitro Cell Dev Biol Anim. 2015 Oct 1;52:77–88. doi: 10.1007/s11626-015-9949-0 (PMC4701759; doi:10.1007/s11626-015-9949-0)
Supplement: Supplementary file 1 — Polymerase chain reaction (PCR) primers and amplification products from B wStr homologs of WD0611–WD0620. Products are depicted schematically in Fig. 2b. (DOCX 116 kb) [file 11626_2015_9949_MOESM1_ESM.docx]

### Supplemental Table S1

Polymerase chain reaction primers and products obtained from ^B^*w*Str homologs of WD0611 – WD0620.

| Gene | Name | Forward primer | Name | Reverse primer | Product bp |
| --- | --- | --- | --- | --- | --- |
| WD0611 | WD0611-F4 | TTGCCCTAGAAAGCCTGGAG | WD0612-GR1 | CGAGAAATTGCAGCCAGATG | 792 |
| WD0611 | WD0611-F5 | TCACAATGCAAAACCGCTGAG | WD0612-GR1 | C GAGAAATTGCAGCCAGATG | 917 |
| WD0612 | WD0612-F5 | ATGCCGCTACTCGCTAAGTG | WD0612-R5 | ATGGGCAAGAGTTTGGCTGT | 852 |
| WD0612 | WD0612GF1 | GTTATTCATGGTTACCCTCC | WD0613GR1 | GCATCTCTGTGCTTAAACAAC | 1106 |
| WD0613 | WD0613-F2 | AACGGCTGATTATGGTGGCA | WD0613-R2 | TTCATCTGGAGCACAGCAGG | 1063 |
| WD0613 | WD0613-F6 | ATTTGGCTCCAGGTGGTCAG | WD0614-R6 | TTCCCACAACGCCCTTAACA | 1132 |
| WD0614 | WD0614-F1 | AGGGCGTTGTGGGAAATAGG | WD0614-R1 | GCAGAAAGCGGCAAAAGACA | 505 |
| WD0614 | WD0614-F2 | TGTCTTTTGCCGCTTTCTGC | WD0615-R2 | CCCCATCTTCCAGTGCAGTT | 442 |
| WD0615 | WD0615-F1 | AACTGCACTGGAAGATGGGG | WD0615-R1 | TGAAGGCTGAGGTTTTGGCA | 522 |
| WD0615 | WD0615-F3 | GTGCCAAAACCTCAGCCTTC | WD0616-R3 | CATGCGCTTGCCGAGAAAAT | 1036 |
| WD0616 | WD0616-GF2 | GCTTAGTACATAAGGTCAGTAGC | WD0616-GR2 | AAGTAGATTTACCACCTCCAG | 789 |
| WD0616 | WD0616-GF3 | ACCATTAATCCTTGCGAAAAGG | WD0617-GR3 | TCAGCAACATCTAAGATTCCG | 1020 |
| WD0617 | WD0617-F1 | GATAGCGCAGGCAATGCAAA | WD0617-R1 | AGCCATCGCTTCCCTCATTT | 848 |
| WD0617 | WD0617-F2 | ACCCATCTTGGCACAAAGGA | WD0618-R2 | ACACAGCTCCACCACCAAAA | 525 |
| WD0618 | WD0618-F4 | TGGAGCTGTGTGGCCTTTAG | WD0618-R4 | CCAGCTTGGTGCATACCTCC | 276 |
| WD0618 | WD0618-F4 | TGGAGCTGTGTGGCCTTTAG | WD0619-GR1 | AATAGGTAGTAAAGTTGGCGC | 1273 |
| WD0619 | WD0619-F2 | ATGAGCGCATCTCAGCAACT | WD0619-R2 | CGAGTGCAACAACAGAGCAA | 905 |
| WD0619 | WD0619-GF2 | GCATCTGCATAGAGCTATTTGG | WD0619-GR2 | GTAAGCATAAGAAACCAGAGTAC | 541 |
| WD0620 | WD0620-F1 | CCCTATAGCATTGTCCGCGT | WD0620-R1 | GCTGGTCCAGGATTTGGAGG | 360 |
| WD0620 | WD0620-F2 | TGCTCTGTTGTTGCACTCGT | WD0620-R2 | ACGCGGACAATGCTATAGGG | 307 |
| WD0620 | WD0620-GF1 | ATGGTACTTTGATATTAATACCATC | WD0620-GR1 | AGCTTAACATTGTGGTCTTCAGC | 973 |
